# Optical frequency domain imaging of the scoring balloon elements shift

**DOI:** 10.1002/ccr3.7550

**Published:** 2023-06-13

**Authors:** Eiji Karashima, Takeo Kaneko

**Affiliations:** ^1^ Department of Cardiology Shimonoseki City Hospital Yamaguchi Japan

**Keywords:** endovascular treatment, optical frequency domain imaging, scoring balloon

## Abstract

Here, we report a case of endovascular treatment in which optical frequency domain imaging evaluated the scoring balloon elements shift when three inflations without shaft rotation performed with a scoring balloon.

## Graphical abstract

Angiography and OFDI of the NSE three inflations.

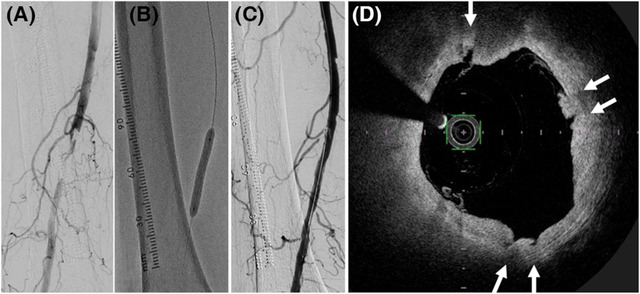

## 1 CASE PRESENTATION

**FIGURE 1:**
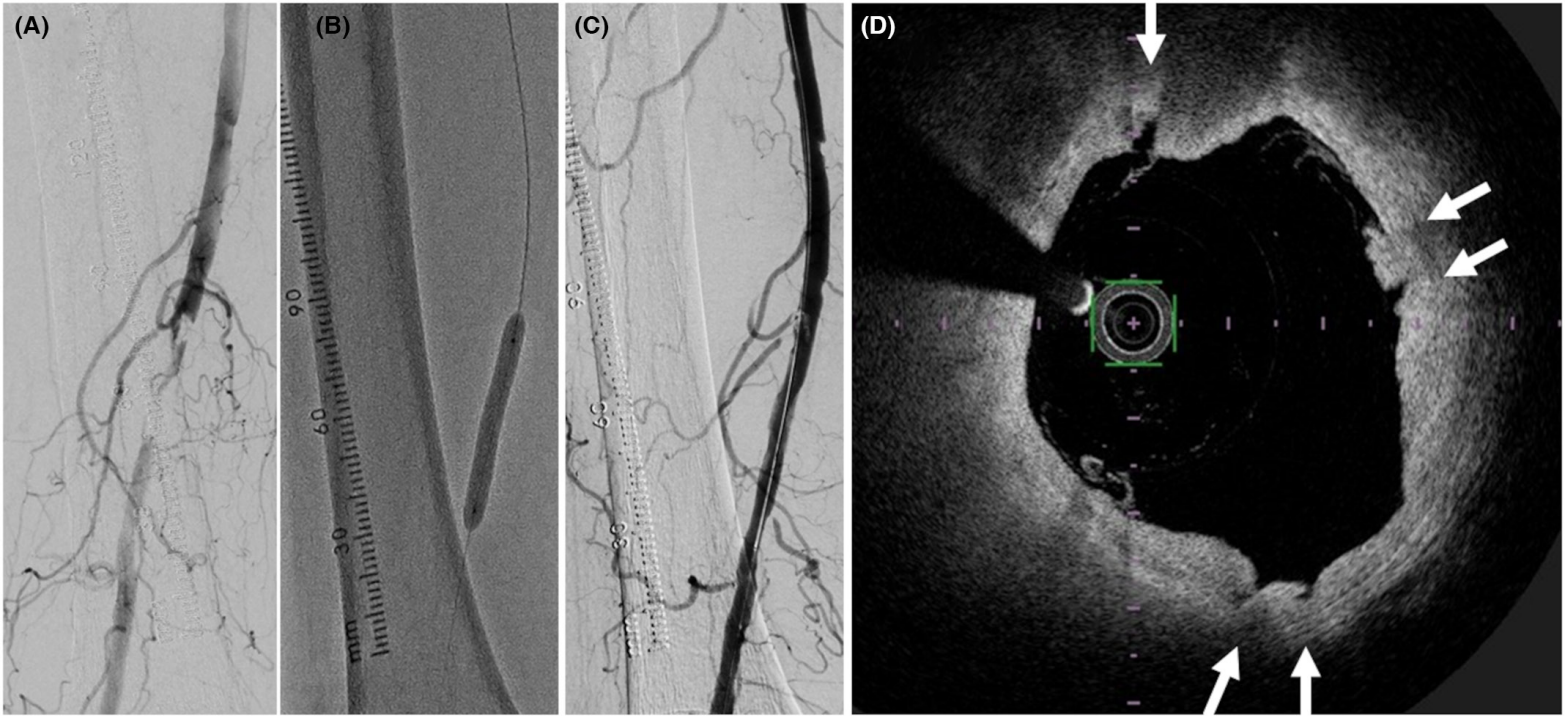
(A–C) Angiography of the endovascular treatment with three inflations of a 5.0 × 40‐mm‐long NSE PTA balloon for the right superficial femoral arterial occlusion. (D) OFDI after the NSE three inflations. Arrows showed the scores induced by a NSE PTA balloon.

Endovascular treatment was performed for an 89‐year‐old male with a symptomatic chronic total occlusion in the right superficial femoral artery (Figure 1A). After crossing the chronic total occlusion with a 0.014‐inch wire, a red clot was collected by catheter thrombectomy. A 5.0 × 40‐mm‐long non‐slip element percutaneous transluminal balloon angioplasty (NSE PTA)—a scoring balloon—(Nipro Corporation) was inflated three times without shaft rotation (NSE three inflations) (Figure 1B). After that, angiography showed no significant dissection (Figure 1C). In addition, five of the scores induced by the NSE PTA balloon were detected by optical frequency domain imaging (OFDI) (Terumo Corporation) (Figure 1D).

When the NSE three inflations performed with a NSE PTA balloon, the elements shift of NSE PTA balloon could detect in vitro experiment.^1^ However, during the study, the elements shift of NSE PTA balloon could not detect in patients with intravascular ultrasound (IVUS) due to the insufficient resolution of IVUS.

The resolution of OFDI is approximately 10 times greater than that of IVUS,^2^ OFDI could detect the scores induced by the NSE PTA balloon. Moreover, OFDI detected five scores after the NSE three inflations. Because the NSE PTA balloon has three elements, the image of OFDI proved the elements shift of the NSE PTA balloon when the NSE three inflations was performed in a patient.

## AUTHOR CONTRIBUTIONS

**Eiji Karashima:** Investigation; writing – original draft. **Takeo Kaneko:** Writing – review and editing.

## FUNDING INFORMATION

This report did not receive any specific funding.

## CONFLICT OF INTEREST STATEMENT

The authors have no conflict of interest to declare.

## CONSENT

Written informed consent was obtained from the patient to publish this report in accordance with the journal's patient consent policy.

## ETHICS STATEMENT

None.

## Data Availability

All data are included in the case report.
